# MLH1 germline mutation associated with Lynch syndrome in a family followed for more than 45 years

**DOI:** 10.1186/s12881-019-0792-0

**Published:** 2019-05-02

**Authors:** Tomoyuki Momma, Kenji Gonda, Yoshinori Akama, Eisei Endo, Daisuke Ujiie, Shotaro Fujita, Yuko Maejima, Shoichiro Horita, Kenju Shimomura, Shigehira Saji, Koji Kono, Rei Yashima, Fumiaki Watanabe, Kokichi Sugano, Tadashi Nomizu

**Affiliations:** 1 Department of Gastrointestinal Tract Surgery, Fukushima Medical University, 1-Hikarigaoka, Fukushima city, Fukushima, 960-1295 Japan; 2 Center for Medical Genetics, Fukushima Medical University, 1-Hikarigaoka, Fukushima city, Fukushima, 960-1295 Japan; 3 Clinical Oncology Center, Fukushima Medical University Hospital, 1-Hikarigaoka, Fukushima city, Fukushima, 960-1295 Japan; 4 Department of Bioregulation and Pharmacological Medicine, Fukushima Medical University, 1-Hikarigaoka, Fukushima city, Fukushima, 960-1295 Japan; 5 Department of Surgery, Hoshi General Hospital, 59-1 Mukaikawahara Koriyama city, Fukushima, 963-8501 Japan; 6 Oncogene Research Unit/Cancer Prevention Unit, Tochigi Cancer Center Research Institute, Younan 4-9-13, Utsunomiya, Tochigi 320-0834 Japan

**Keywords:** Lynch syndrome, MLH1 germline mutation

## Abstract

**Background:**

Lynch syndrome, is an autosomal dominantly inherited disease that predisposes individuals to a high risk of colorectal cancers, and some mismatch-repair genes have been identified as causative genes. The purpose of this study was to investigate the genomic rearrangement of the gene in a family with Lynch syndrome followed for more than 45 years.

**Case presentation:**

The family with Lynch syndrome is family N, who received colorectal cancer treatment for 45 years. The proband of family N had multiple colorectal and uterine cancers. Because the proband met the diagnostic Amsterdam criteria and was Microsatellite instability (MSI) - positive, we performed genetic testing several times. However, germline mutations in *MLH1* and *MSH2* genes were not found by long-distance PCR or RT-PCR/direct sequencing analysis within the 45-year follow-up. MLPA analysis showed that the genomes of the proband and proband’s daughter contained a deletion from exon 4 through exon 19 in the *MLH1* gene. Her son’s son and her daughter’s son were found to be carriers of the mutation.

**Conclusions:**

For carriers of mismatch-repair gene mutation among families with Lynch syndrome, the onset risk of associated cancers such as uterine cancer is particularly high, including colorectal cancer. The diagnosis of carriers among non-onset relatives is important for disease surveillance.

## Background

Lynch syndrome (LS) is an autosomal dominantly inherited syndrome that is caused by germline mutations of DNA- mismatch repair genes, such as *MLH1*, *MSH2*, *MSH6* and *PMS2*. Most of these mutations have been detected in the *MLH1* and *MSH2* genes [1–8]. Patients with LS must fulfil the Amsterdam criteria [9], and MSI is a hallmark of most of the cancers associated with LS. In *MLH1* and *MSH2* mutation carriers, MSI has been found in > 90% of colorectal cancers (CRCs). By using conventional methods of mutation analysis, point mutations in the DNA mismatch repair genes *MLH1* and *MSH2* have been detected in up to 60% of patients suspected of having LS. In addition, more than 90% of the mutations detected in family members with LS were in either *MLH1* or *MSH2* [10–13]. However, large genomic deletions cannot be detected by these methods. That is, if all genomes are not widely screened, it is difficult to understand the gene deficiency accurately. Approximately 50–60% of genetic mutations are detected in LS. Here, we assessed the performance of MLPA (MRC-Holland, Amsterdam, and the Netherlands) as an alternative method for the detection of genomic deletions in the *MLH1* and *MSH2* genes. This method is a quantitative multiplex PCR approach to determine the relative copy number of each exon in a given gene. The MLPA approach has proven to be very useful for the screening of large numbers of LS patients harbouring exonic deletions [14]. We screened for the genomic rearrangement of the *MLH1* and *MSH2* genes in this family, whose members fulfilled the Amsterdam criteria but were negative for genetic mutations by conventional diagnostic methods. We identified a germline mutation in the *MLH1* gene with the gene rearrangement of an 89,081-bp region from exon 4 through exon 19 of the *MLH1* gene in two related patients. In addition, the breakpoint, assumed to be a cause of the gene rearrangement, was analysed in family members with *MLH1* genetic abnormalities.

### Case presentation

#### Clinical information

**Fig. 1:**
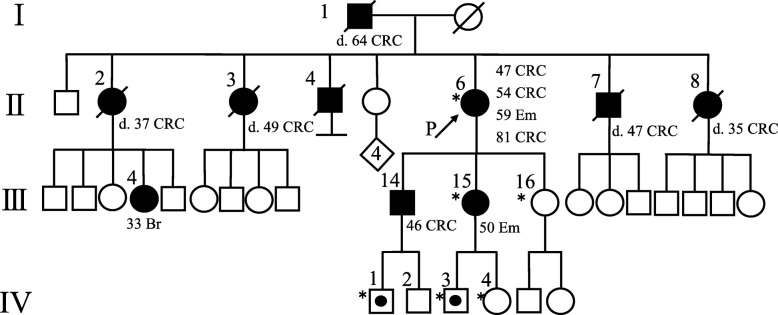
Family pedigree. The reconstructed pedigree shows that the proband (II-6), her son's son (VI-1), her daughter (III-15) and her daughter's son (IV-3) share the mutation. I-1 T-colon cancer. II-2 A-colon cancer. II-3 T-colon cancer. II-4 primary cancer unknown, age unknown. II-6 rectal cancer, sigmoid cancer, endometrial cancer and rectal cancer. II-7 A-colon cancer. II-8 caecal cancer. III-4 breast cancer. III-14 A-colon cancer and sigmoid cancer. III-15 Endometrial cancer. II-6, III-15, III-16, IV-1, IV-3 and IV-4 underwent genetic testing. IV-1 and IV-3 were found to be mutation carriers. Squares denote male family members, circles denote female family members, solid symbols show individuals affected by cancer, the arrow denotes the proband, symbol with a slash shows a deceased person with the age at death, and types of primary tumours are listed below the symbols. Solid circle in square shows mutation carrier. P: proband, CRC: colorectal cancer, Em: endometrial cancer, Br: breast cancer, *: genetic testing was performed

An 81-year-old female (Proband, patient II-6) had rectal cancer (at 47 years of age), sigmoid cancer (at 54 years of age), endometrial cancer (at 59 years of age) and rectal cancer (at 81 years of age). Her son (patient III-14) had A-colon cancer (at 46 years of age). Her daughter (patient III-15) had endometrial cancer (at 50 years of age). Her three sisters had A-colon cancer (at 33 years of age, patient II-2) and was deceased (at 37 years of age), T-colon cancer (at 47 years of age, patient II-3) and was deceased (at 49 years of age) and A-colon cancer (at 34 years of age, patient II-8) and was deceased (at 35 years of age). Her brother had caecal cancer (at 35 years of age, patient II-7) and was deceased (at 47 years of age). Her father had T-colon cancer (at 60 years of age, patient I-1) and was deceased (at 64 years of age). Her sister’s daughter had breast cancer (at 33 years of age, patient III-4) (Fig. 1). MLPA analysis was performed in patients who were referred to genetic counselling clinics at the Hoshi General Hospital. Heparinized peripheral blood lymphocytes were collected from the proband and her daughter and analysed for large genomic disorganization of the *MLH1* gene. The protocol was approved by the Ethical Review Board of the Hoshi General Hospital and conformed to the ethical guidelines on human genome studies. Additional informed consent was obtained from all individual participants for whom identifying information was included in this article. According to the genetic screening and test, the approval of the Ethical Review Board was obtained in all families.

### Lynch syndrome criteria and pedigree profiles

This family fulfilled the Amsterdam criteria, the revised Amsterdam II criteria and the Bethesda guidelines for the diagnosis of LS [15, 16]. In 1990, the International Collaborative Group reported the following minimum diagnostic criteria (Amsterdam criteria). (1) At least three relatives should have histologically verified CRCs, one of whom should be a first-degree relative to the other two members. Familial adenomatous polyposis should be excluded. (2) At least two successive generations should be affected. (3) CRC should be diagnosed in at least one of the relatives at an age younger than 50 years.

### MSI analysis

For the proband and her daughter, tissues from tumour and corresponding normal mucosa tissues were obtained from two paraffin-embedded tumours (two colon lesions), each to analyse MSI. A high frequency of MSI was shown in all tumours, suggesting MMR (mismatch repair genes) deficiency. We subsequently performed PCR analysis at 13 microsatellite repeat loci, of which 5 loci we are compatible with the Bethesda panel (BAT25, BAT26, D2S123, D5S346 and D17S250) and 8 mononucleotide repeat loci we are reported to show MSI [BAX, TGFβRII, MSH3, MSH6, PTEN exon 7, PTEN exon 8, MBD4 (A)6 and MBD4 (A)10] with relatively high frequencies.

### MLPA analysis

**Fig. 2:**
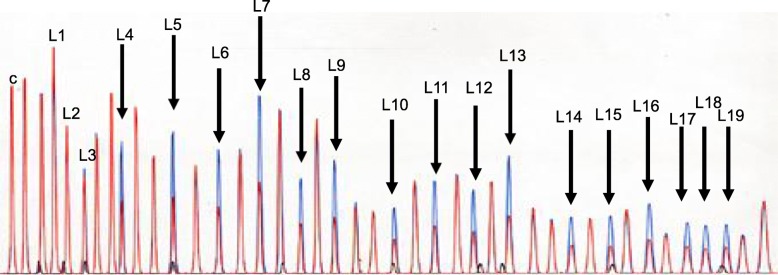
MLPA analysis in family N. Detection of the *MLH1* exons 4-19 deletion by MLPA. The panel shows electropherograms and results of the MLPA analyses. Profiles corresponding to *MLH*1 exons and control probes were obtained from the overlap of a control sample (blue) with a proband sample (red). The numbers in the top figure refer to *MLH1* exons recognized by each MLPA probe. The arrowheads and numbers show the deleted *MLH1* exons. “c” indicates control peaks resulting from the amplification of probes located in different chromosomes. Deletions are detected by a 0.5-fold decrease in the peak height compared with that of a normal control. MLPA results demonstrated a decrease of peaks corresponding to *MLH1* exons 4-19

We used probe mix P003 (MRC Holland) for MLPA, which contains 40 sets of probes that hybridize to the 19 exons in *MLH1*; 7 control probes of other human genes located on different chromosomes are included as controls (Fig. 2). Details on probe sequences can be found on the manufacturer’s website (http://www.mrc-holland.com).

MLPA was performed according to the manufacturer’s instructions. Data analyses were performed with the Gene Scan 3.7 software. The results from Gene Scan were exported to Excel, where the final results were calculated.

### Mutation analysis

**Fig. 3:**
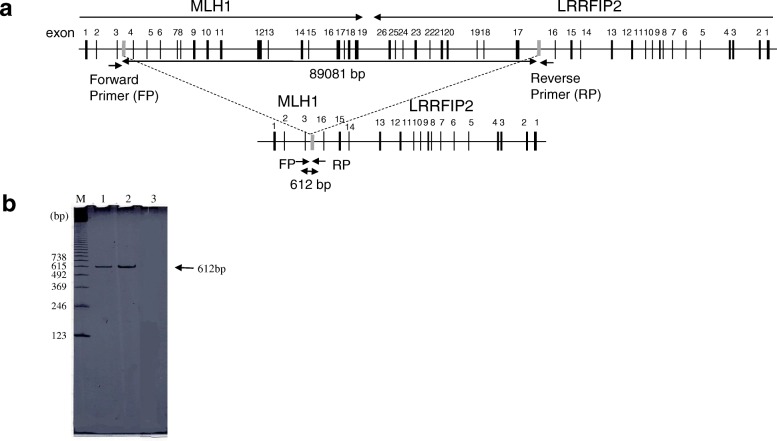
*MLH1* deletion site and PCR. The breakpoint for genomic deletion was detected by breakpoint-specific PCR. Schematic illustration of the wild-type and mutant alleles of *MLH1* genomic deletions. Exons are numbered. The grey line indicates the recombination site. **a**. The sequence of the forward PCR primer (FP) was 5′-TTTAGCCAAGTATTTCTACCTATGG-3′ and designed in *MLH1* intron 3, while that of the reverse primer (RP) was 5′-TCAAGCCTCCTGTTATGAAGA-3′ and designed in *LRRFIP2* intoron 16. **b**. An amplified DNA fragment of 612 bp was obtained in the analyses of genomic DNA of the proband (lane 1) and her daughter (lane 2), while no amplification products were obtained from normal genomic DNA (lane 3). M: molecular weight marker ladder

**Fig. 4:**
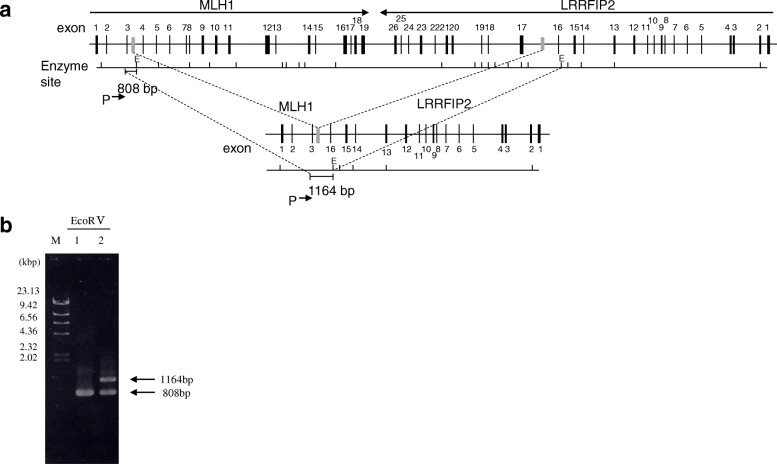
Restriction EcoRV enzyme digestion and PCR. **a**. Schematic illustration of the wild-type and mutant alleles of *MLH1*. Exons are numbered. The location of the intronic restriction sites (E, EcoRV) is indicated. The gray line indicates the recombination site. Primary PCR was performed with the gene-specific primer 5′-AGAAAGAAGATCTGGATATTGTATGTGA-3′. The short line indicates the normal allele obtained by restriction enzyme digestion. P: primer. **b**. The PCR product obtained by restriction enzyme digestion of the wild-type allele was 808 bp (lane 1). The PCR product obtained by restriction enzyme digestion of the mutant allele was 1164 bp (lane 2). M: molecular weight marker ladder

**Fig. 5:**
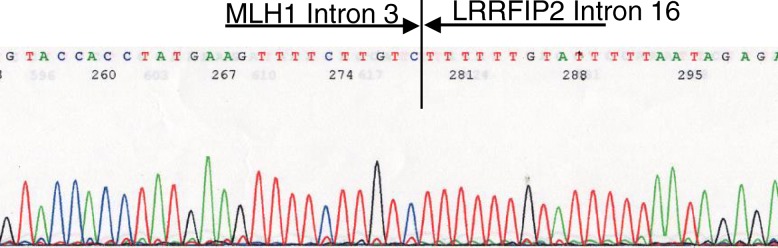
PCR/direct sequencing analysis of genomic DNA. Sequence of the PCR product showing the breakpoint region within *MLH1* intoron 3 and *LRRFIP2* intron 16 of the junction fragment. Characterization of the deletion breakpoint, indicated that the *MLH1* structural abnormal genes are a *MLH1* 4-19 deletion, nucleotides: 129,810 - 118,893, size: 89,083 bp

We detected large deletions of *MLH1* exons 4-19 in this family (c. (306 + 1_307–1)_(*193_?) del., InSiGHT classification: Class 5) by MLPA assay (Fig. 2). To determine the breakpoint for this genomic rearrangement, we performed PCR using multiple sets of forward and reverse primers, for which the forward primers were designed in intron 3 of *MLH1* and the reverse primers were designed in intron 16 of leucine-rich repeat interacting protein 2 (*LRRFIP2*) (Fig. 3a). PCR products were sequenced on an ABI Prism 310 genetic analyser using 310 Gene Scan 2.11 software. PCR using this primer set did not amplify a DNA fragment in genomic DNA from normal mucosa, while a 612-bp DNA fragment was amplified from genomic DNA in both tissues from the proband (Fig. 3b, lane 1). A PCR product of a similar 612-bp size was obtained from her daughter (Fig. 3b, lane 2). PCR analysis of a close relative with the same primer set indicated that member was a carrier of the mutation. Restriction enzyme digestion (EcoRV) and PCR with a primer targeting *MLH1* exon 3 yielded PCR products of the control sample (Fig. 4b, lane 1), which were and 808 bp in size, and for the patient sample (Fig. 4b, lane 2), which were 1164 bp in size. Direct sequencing analysis revealed that the rearrangement site was located approximately 261 bp downstream of exon 3 (Fig. 5). Her other daughter, her son’s son, her daughter’s son and her daughter’s daughter underwent genetic testing. Her son’s son and her daughter’s son were revealed to be mutation carriers (Fig. 1).

## Discussion and conclusions

This deletion of *MLH1* exons 4-19 has been reported according to the International Society for Gastrointestinal Hereditary Tumours Database (https://www.insightgroup.org/variants/databases/), and the International Collaborative Group on HNPCC Database (http://www.insight-group.org/). However, this mutation has been reported in SKOV-3 cells [17]. We detected mutations in four members of a family with Lynch syndrome. The MLPA assay proved to be robust and reliable in most cases as seen by even peak heights across the multiplex PCR. Moreover, it allows for prompt screening compared with conventional diagnostic techniques, as many exons can be evaluated in a single run, leading to the development of an inspection system. By MLPA assay, we found both deletions of *MLH1* exons 4-19 (c.(306 + 1_307–1)_(*193_?) del.) and *MSH2* exon7 (c. (1076 + 1_1077–1)_(1276 + 1_1277–1) del. p.Leu360Lysfs*16) in other MSI -positive families with suspected Lynch syndrome. Furthermore, we found deletions of *MSH2* exons 7-14 (c. (1076 + 1_1077–1)_(2458 + 1_2459–1)del) in another family. In these 2 families, for dozens of years, the germline mutation was not identified by the conventional assay. Because mutations were identified, the members of these families have received personalized and precision medicine. In an autosomal dominantly hereditary disorder that shows imperfect infiltration such as causative mutations in LS, individuals in the pedigree with many genetic mutations must be identified and provided prompt screening of genetic abnormalities. The screening of the cancers such as genetic panel examinations will be performed widely in the future. The somatic mutations of genes such as *MLH1*, and *MSH2 * will be found as secondary findings and accidental findings. At that time, we confirm a family history and should discover germline mutations quickly.

## Abbreviations

LS: Lynch syndrome
MLPA: Multiplex ligation-dependent probe amplification
